# Novel astrocyte targets: New Avenues for the Therapeutic Treatment of Epilepsy

**DOI:** 10.1177/1073858414523320

**Published:** 2015-02

**Authors:** Vincenzo Crunelli, Giorgio Carmignoto, Christian Steinhäuser

**Affiliations:** 1 Neuroscience Division, School of Biosciences, Cardiff University, Cardiff, UK; 2 Centro Nazionale della Ricerca, Neuroscience Institute and Department of Biomedical Sciences, University of Padova, Padova, Italy; 3 Institute of Cellular Neurosciences, Medical Faculty, University of Bonn, Bonn, Germany

**Keywords:** glutamate, GABA, connexin, potassium, temporal lobe epilepsy, absence epilepsy

## Abstract

During the last 20 years, it has been well established that a finely tuned, continuous crosstalk between neurons and astrocytes not only critically modulates physiological brain functions but also underlies many neurological diseases. In particular, this novel way of interpreting brain activity is markedly influencing our current knowledge of epilepsy, prompting a re-evaluation of old findings and guiding novel experimentation. Here, we review recent studies that have unraveled novel and unique contributions of astrocytes to the generation and spread of convulsive and nonconvulsive seizures and epileptiform activity. The emerging scenario advocates an overall framework in which a dynamic and reciprocal interplay among astrocytic and neuronal ensembles is fundamental for a fuller understanding of epilepsy. In turn, this offers novel astrocytic targets for the development of those really novel chemical entities for the control of convulsive and nonconvulsive seizures that have been acknowledged as a key priority in the management of epilepsy.

## Astrocyte-Neuron Reciprocal Signaling

Being unable to generate action potentials, astrocytes were for a long time considered nonexcitable cells. However, in studies from the 1990s, a Ca^2+^-based form of excitability underlying long-distance communication between astrocytes in cultures was revealed by the observation that Ca^2+^ increases evoked in individual astrocytes by the neurotransmitter glutamate could propagate to neighboring astrocytes as an intercellular Ca^2+^ wave (Charles and others 1991; Cornell-Bell and others 1990; Finkbeiner 1992). This pioneering finding challenged the common belief that astrocytes merely act in the brain as neuron-supportive elements and paved the way for a new era in astrocyte research. Studies in brain slice preparations then reported that astrocytes respond to the synaptic release of glutamate (Pasti and others 1997; Porter and McCarthy 1996), GABA (Kang and others 1998), noradrenaline (Duffy and MacVicar 1995; Kulik and others 1999), and acetylcholine (Araque and others 2002; Navarrete and others 2012; Shelton and McCarthy 2000; Takata and others 2011) with complex, often repetitive cytosolic Ca^2+^ elevations, which primarily depend on the activation of G protein–coupled metabotropic receptors (Verkhratsky and Kettenmann 1996; Verkhratsky and others 1998) linked to phospholipase C, production of inositol 1,4,5-trisphosphate (InsP3), activation of InsP3 receptors, and finally, release of Ca^2+^ from intracellular Ca^2+^ compartments (Berridge 1993; Haydon and Carmignoto 2006; Pozzan and others 1994; Shigetomi and others 2011). Brain slice results were then validated in in vivo experiments that used imaging of fluorescence Ca^2+^ signals by two-photon laser scanning microscopy in anesthetized as well as awake, freely moving mice. By using this new in vivo approach, Hirase and colleagues (2004) could reveal neuronal activity–evoked Ca^2+^ elevations in the large majority of cortical astrocytes. Correlated Ca^2+^ increases between adjacent astrocytes were also detected, suggesting that in the neuron-astrocyte network, in vivo neurons can signal simultaneously to multiple astrocytes. Subsequent studies confirmed that astrocytes from different brain regions, including the olfactory bulb (Petzold and others 2008), cerebellum, and neocortex (Schummers and others 2008; Wang and others 2006), can respond to sensory stimulation with complex Ca^2+^ elevations. The amplitude, frequency, kinetics, and also spatial extent of astrocyte Ca^2+^ elevations were revealed to be strictly dependent on the pattern and strength of neuronal activity (Pasti and others 1997; Todd and others 2010), suggesting an astrocyte encoding of neuronal information (Bezzi and others 1998; Kang and others 1998; Pasti and others 1997).

**Figure 1.**
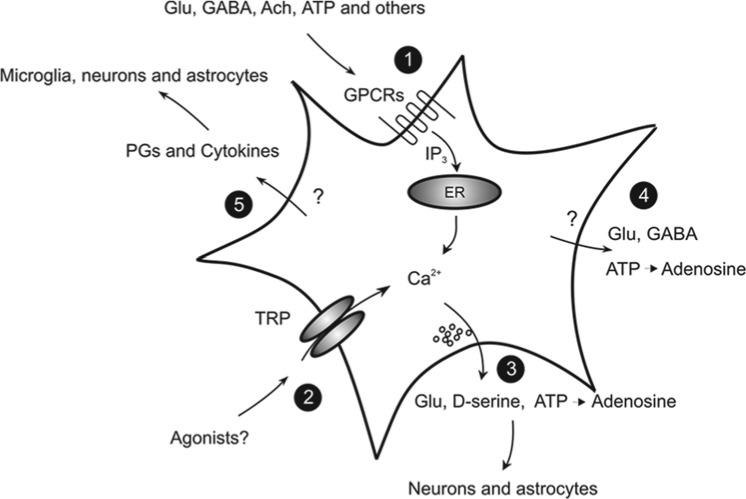
Regulated release of gliotransmitters that can affect epileptiform activities. (1) Classic neurotransmitters activate G protein–coupled receptors (GPCRs) and cytosolic Ca^2+^ elevations in astrocytes. (2) Transient receptor potentials, such as TRPA1, mediate Ca^2+^ influx and contribute to cytosolic Ca^2+^ regulation. The gliotransmitters that contribute to the regulation of neural network excitability, such as glutamate, D-serine, GABA, and ATP, are released via both Ca^2+^-dependent (3) and Ca^2+^-independent (4), nonmutually exclusive mechanisms. (5) Other signaling molecules, including inflammatory mediators, such as prostaglandins (PGs) and diverse cytokines, are also released, but through undefined mechanisms. By targeting microglia, neurons, and also astrocytes, these molecules can enhance seizure generation.

**Figure 2.**
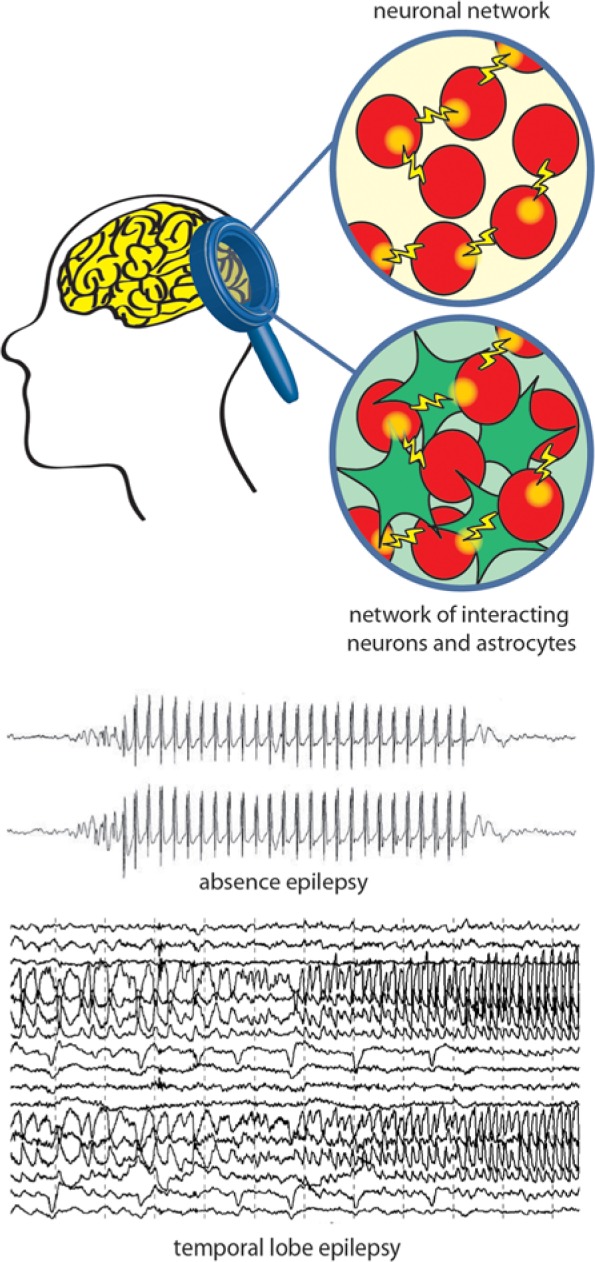
A schematic drawing of the abnormal electrical activity during epileptic seizures. Absence and temporal lobe seizures (see electroencephalogram traces) originate from malfunctions in neurons (classic view, top circle) or in the finely tuned crosstalk between astrocytic (green) and neuronal (red) networks (novel view, bottom circle).

These studies proved beyond a doubt that neurons “excite” astrocytes through the same signals that transfer information to postsynaptic neurons. What is most relevant for the topic of this article is that this “neuronal excitation” promotes in astrocytes either a feedback or a feedforward signal or both that can take the form of a Ca^2+^-dependent release of diverse neuroactive molecules, the so-called gliotransmitters, which contribute to the control of neuronal network excitability (Fig. 1). Among these are classic chemical transmitters, such as glutamate (Bezzi and others 1998; Parpura and others 1994), D-serine (Henneberger and others 2010; Zhuang and others 2010), ATP (Zhang and others 2003), (Bowser and Khakh 2004) and GABA (Kozlov and others 2006; Le Meur and others 2012), but also vasoactive agents, such as prostaglandins, and inflammatory signals, such as interleukin-1β (IL-β), high-mobility group box 1 (HMGB1), and tumor necrosis factor α (Bezzi and others 2001; Maroso and others 2010; Vezzani and others 2008). The regulated, activity-dependent release of different gliotransmitters has been shown 1) to modulate short- and long-term changes in synaptic transmission (Brockhaus and Deitmer 2002; Henneberger and others 2010; Jourdain and others 2007; Panatier and others 2006; Pascual and others 2005; Pasti and others 1997; Perea and Araque 2007; Serrano and others 2006; Shigetomi and others 2011; Zhang and others 2003), 2) to control cerebral blood flow (Haydon and Carmignoto 2006; Petzold and others 2008; Zonta and others 2003), 3) to favor neuronal synchrony in the neuronal network (Angulo and others 2004; Fellin and others 2004), and 4) to contribute to brain inflammatory and immune responses (Aronica and others 2012; Hamby and Sofroniew 2010) (Fig. 1). While our effort in the evaluation of the richness in gliotransmitter modulatory actions in the brain leads to a new understanding of the astrocyte’s role in brain function, we are also beginning to realize how deep and wide the impact of gliotransmission might be on the pathogenesis of different brain disorders, including epilepsy (Fig. 2).

## Astrocytes and Epilepsy

Epilepsy is a neurological disease that is characterized by the periodic occurrence of spontaneous seizures, which can be convulsive or nonconvulsive in nature depending on the type of epilepsy (Noebels and others 2012). Among the most common and severe forms of convulsive epilepsies is temporal lobe epilepsy (TLE). The clinical manifestation of this disorder is represented by recurrent seizures that arise as an intense, synchronous discharge in a relatively high number of neurons from a restricted region of the medial (mTLE) or lateral temporal lobe, that is, the epileptogenic focus, and eventually generalize to both temporal lobes and extratemporal structures through a progressive recruitment of other neuronal populations (Avoli and others 2002; Cammarota and others 2013; Pinto and others 2005; Traub and Wong 1982; Trevelyan and others 2006). As we mentioned above, through the release of different gliotransmitters, astrocytes contribute to the regulation of both neuronal excitability and synaptic transmission and favor neuronal synchrony (Fellin and others 2004). The reactive gliosis revealed from neurosurgical specimens of patients presenting with mTLE may have an impact on this astrocyte signaling, leading to an increase in local network excitability and synchronous discharges that predispose neurons to generate focal epileptiform activities in TLE. In support of this view, various membrane channels, receptors, and transporters in astrocytic membranes are altered in the epileptic brain. Excitingly, recent evidence, reviewed below, suggests that in the course of the pathogenesis of mTLE, these glial changes alter homeostatic network functions, temporally precede alterations in neurons, and thus seem to be causative for the disorder.

At the other end of the clinical spectrum of epilepsies are the nonconvulsive absence seizures, which consist of sudden and brief periods of impairment of consciousness that are always accompanied by generalized and synchronous spike and wave discharges on electroencephalograms (Avoli 2012; Blumenfeld 2005; Crunelli and Leresche 2002; Panayiotopoulos 1997). Absence seizures are present, either alone or most commonly in association with convulsive seizures, in many idiopathic generalized epilepsies and are known to be generated by paroxysmal activity within cortical and thalamic networks with little or no involvement of other brain regions (Bai and others 2010; Crunelli and Leresche 2002; Vergnes and Marescaux 1992; Williams 1953). Differently from convulsive seizures, clinical evidence indicates that drugs that increase GABAergic function aggravate and induce absence seizures in patients with absence seizures and in healthy individuals, respectively (Ettinger and others 1999; Panayiotopoulos 2001; Perucca and others 1998). Thus, an increased GABAergic function may be an underlying cause of these seizures: indeed, it has been demonstrated that the enhancement of tonic GABA_A_ receptor function in the thalamus of genetic absence epilepsy models is due to a malfunction of the astrocytic GABA transporter GAT-1 (Cope and others 2009).

In this review, we summarize the current evidence of astrocyte dysfunctions in epilepsy and discuss potential underlying mechanisms (see also Sifert and others 2006, 2010). Specifically, we discuss how reciprocal signaling between neurons and astrocytes at the level of local brain circuits may contribute to the generation of epileptogenic foci in mTLE models. We also clearly demonstrate a critical role of astrocytes in the disturbance of K^+^ and transmitter homeostasis, as well as GABA transporter function and cytokine regulation, and the impact that these abnormalities have on the generation of both convulsive and nonconvulsive seizures. Thus, although research on astrocytes in epilepsy is still in its infancy, these findings might eventually lead to a classification of mTLE and absence seizures as disorders of astrocyte-neuron signaling rather than neuronal diseases. Importantly, because currently available anticonvulsant drugs and therapies are insufficient to control mTLE and absence seizures in about one third of patients, ongoing research on astrocytic abnormalities in epilepsy is providing promising new astrocytic targets, which may lead to more specific antiepileptogenic therapeutic strategies.

## K Channels, Gap Junctions, and Aquaporins in mTLE

### K^+^ Uptake and K^+^ Spatial Buffering

**Figure 3.**
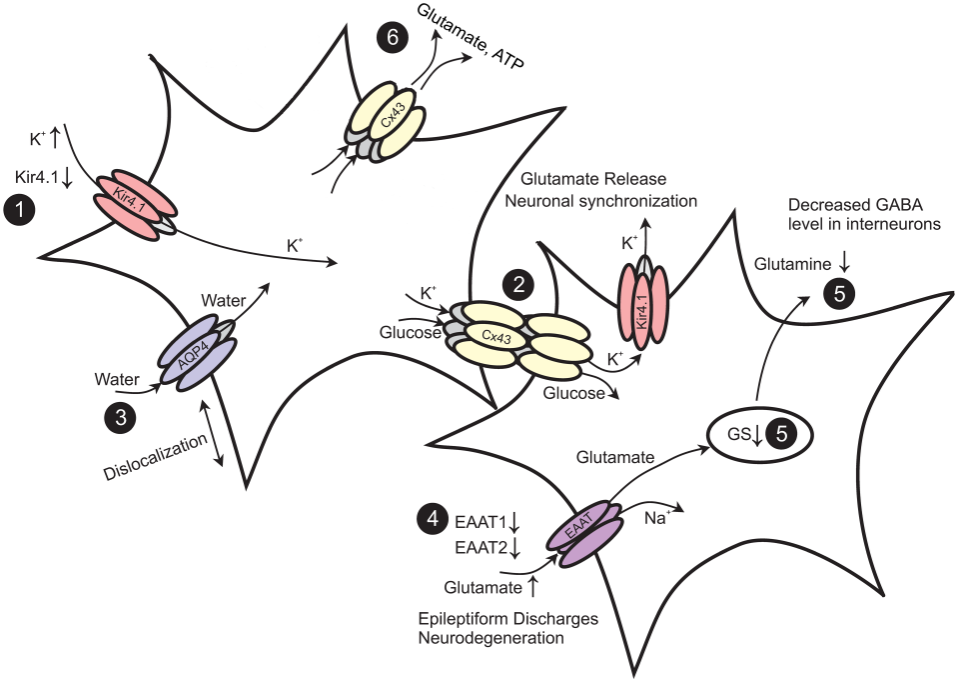
Epilepsy-associated alterations of functional properties in astrocytes. (1) Seizure activity leads to an increase in extracellular K^+^ concentration. Down-regulation of Kir channels was observed in astrocytes in human and experimental epilepsy. (2) Gap junctions (GJs) mediate the spatial redistribution of K^+^. Genetic ablation of GJs entails impaired K^+^ buffering and hyperactivity through neuronal depolarization-induced glutamate release and synchronization. (3) The dislocation of water channels contributes to impaired K^+^ buffering. (4) Astrocytes are primarily responsible for glutamate uptake. Reduction of the EAAT1 and EAAT2 proteins is observed in the human epileptic hippocampus. Elevated extracellular glutamate decreases the threshold for seizure induction. (5) Glutamate is converted into glutamine through glutamine synthetase (GS). In the human epileptic hippocampus, loss of GS resulted in elevated extracellular glutamate levels. In experimental epilepsy, down-regulation of GS was observed in the chronic phase. (6) The release of glutamate and ATP from astrocytes through hemichannels may promote hyperexcitability. Modified and reproduced with permission from Steinhäuser and Seifert (2012).

Neuronal activity elicits transient increases in the extracellular potassium concentration ([K^+^]_o_), which under pathological conditions like epilepsy can reach up to 12 mM (Heinemann and Lux 1977). Even moderate rises in [K^+^]_o_ increase neuronal excitability and synaptic transmission (Walz 2000), underscoring the necessity of tight control of K^+^ homeostasis for normal brain function. This task is mainly accomplished by astrocytes, which display very negative resting potentials due to a high resting permeability for K^+^. Responsible for this property are inwardly rectifying K^+^ channels of the Kir4.1 subtype (Seifert and others 2009) (Fig. 3). Astrocytes control [K^+^]_o_ by two mechanisms: K^+^ uptake and spatial buffering. Net uptake of K^+^ is mainly mediated by Na^+^/K^+^ pumps and Na^+^/K^+^/Cl^–^ co-transporters (Kofuji and Newman 2004). It is rather unlikely that this mechanism alone is sufficient for efficient clearance of excess [K^+^]_o_ because intracellular K^+^ accumulation results in water influx and cell swelling. The spatial buffering model (Orkand and others 1966) describes another more effective mechanism for [K^+^]_o_ clearance. It is based on the fact that astrocytes are electrically connected to each other via gap junction (GJ) channels to form a functional syncytium (Fig. 3). According to the spatial buffering model, excessive extracellular K^+^ is taken up by astrocytes at sites of high neuronal activity, redistributed through the astrocytic network, and released at regions of lower [K^+^]_o_. This uptake and release of K^+^ occur passively, driven by the respective electrochemical gradients. Kir4.1 channels are particularly well suited for this task because they possess a high open probability at rest and their conductance increases at high [K^+^]_o_.

### Kir4.1 Channels and K^+^ Buffering in Epilepsy

Increased [K^+^]_o_ has been associated with the pathophysiology of epilepsy (Fisher and others 1976), and it is known that high [K^+^]_o_ is sufficient to trigger epileptiform activity in vitro (Traynelis and Dingledine 1988). To assess the impact of Kir4.1 channels in K^+^ buffering, the effect of Ba^2+^-induced Kir channel block on stimulus-triggered rises in [K^+^]_o_ or iontophoretically applied K^+^ was analyzed. In these experiments, Ba^2+^ enhanced [K^+^]_o_ accumulation under control conditions, but not in sclerotic hippocampi, indicating the disturbance of Ba^2+^-sensitive K^+^ uptake in sclerosis (Kivi and others 2000). This hypothesis was confirmed with patch clamp analysis, demonstrating reduced Kir currents in the sclerotic CA1 region of specimens from patients with mTLE (Bordey and Sontheimer 1998; Hinterkeuser and others 2000). Western blot (Das and others 2012) and immunohistochemistry (Heuser and others 2012) also revealed a significant loss of the Kir4.1 protein in human hippocampal sclerosis (HS). These studies imply that impaired K^+^ clearance and increased seizure susceptibility in mTLE-HS result from the reduced expression of Kir4.1 channels. However, it remains unclear whether this reduction represents a cause, effect, or adaptive response in TLE. In favor of a causative role, David and colleagues (2009) showed in an albumin model of epilepsy that Kir4.1 down-regulation occurs before the onset of epileptic activity. However, another study performed in a kainate model found no changes in astrocytic Kir currents in post–status epilepticus (SE) (Takahashi and others 2010).

Further support for a crucial role of Kir4.1 in glial K^+^ buffering emerged from Kir4.1 knockout mice. Global deletion of Kir4.1 resulted in motor impairments and premature death (Neusch and others 2001). Mice with glia-specific deletion of Kir4.1 (cKir4.1^–/–^ mice) displayed a similarly severe phenotype and demonstrated that loss of Kir4.1 causes epilepsy (Chever and others 2010; Haj-Yasein and others 2011).

Missense variations in *KCNJ10*, the gene encoding Kir4.1, have been linked to seizure susceptibility in man (Buono and others 2004). Loss-of-function mutations in *KCNJ10* underlie an autosomal recessive disorder characterized by seizures, ataxia, sensorineural deafness, mental retardation, and tubulopathy (EAST/SeSAME syndrome) (Bockenhauer and others 2009; Scholl and others 2009). Patients suffering from this disorder display focal and generalized tonic-clonic seizures since childhood. Heuser and colleagues (2010) showed that a combination of three single nucleotide polymorphisms (SNPs) in the *AQP4* gene (encoding a water channel), together with two SNPs in the *KCNJ10* gene, was associated with mTLE. Association analysis in patients with a history of febrile seizures (mTLE-FS) revealed that a combination of SNPs in *KCNJ10, AQP4*, and the area between *KCNJ10* and *KCNJ9* was associated with mTLE-FS (Heuser and others 2012).

### Potential Roles of GJs in Epilepsy

Astrocytes in the adult brain are connected to each other via GJ channels (Fig. 3) composed of connexin 43 (Cx43) and Cx30 (Nagy and Rash 2000), allowing the intercellular exchange of ions, second messengers, metabolites, and amino acids. The astroglial syncytium has important functions, including spatial buffering of K^+^ ions(see above), delivery of energetic metabolites to neurons (Giaume and others 1997), intercellular propagation of Ca^2+^ waves (Scemes and Giaume 2006), volume regulation (Scemes and Spray 1998), and adult neurogenesis (Kunze and others 2009).

The role of interastrocytic coupling in the development and progression of epilepsy is still controversial (Carlen 2012; Nemani and Binder 2005; Steinhäuser and others 2012). According to the spatial buffering concept (see above), the astroglial network is expected to possess antiepileptic function because a reduction of astrocytic coupling would result in the accumulation of extracellular K^+^, neuronal depolarization, and a lowered threshold for seizure generation. In line with this hypothesis are results from mice with coupling-deficient astrocytes. In these mice, clearance of K^+^ and glutamate was disturbed. They displayed spontaneous epileptiform events, a reduced threshold for the generation of epileptic activity, and increased synaptic transmission (Pannasch and others 2011; Wallraff and others 2006). Although these findings strongly support an anticonvulsive role of glial GJ networks, a potential seizure-promoting role emerged from the results by Rouach and colleagues (2008), who demonstrated that astroglial GJs mediate activity-dependent intercellular trafficking of metabolites from blood vessels to sites of high energy demand. The trafficking seemed to be essential for the maintenance of synaptic activity under pathological conditions such as epilepsy. The involvement of GJ channels in the intercellular spread of Ca^2+^ waves also would favor a proconvulsive role of the syncytium through neuronal synchronization and spread of ictal activity (Gomez-Gonzalo and others 2010). Thus, astroglial GJ networks might play a dual, proepileptic and antiepileptic, role in epilepsy. Future work will elucidate which of the mechanisms prevail under various circumstances.

### Cx Expression and Coupling in Epileptic Tissue

Seizure-induced changes in Cx expression have been observed in a variety of animal models and human tissue. The results are conflicting and do not allow us to draw definitive conclusions. In experimental epilepsy, increased (Condorelli and others 2002; Gajda and others 2003; Mylvaganam and others 2010; Samoilova and others 2003; Szente and others 2002; Takahashi and others 2010), unchanged (Khurgel and Ivy 1996; Li and others 2001; Söhl and others 2000; Xu and others 2009), and decreased (David and others 2009; Elisevich and others 1997a, 1998; Xu and others 2009) Cx43 and/or Cx30 transcript and/or protein have been reported. This inconsistency might be explained by differences between animal models, seizure duration, and investigated brain areas. In human specimens, mostly up-regulation of the Cx43 transcript and/or protein has been described (Aronica and others 2001; Collignon and others 2006; Fonseca and others 2002; Naus and others 1991), although unchanged levels have also been reported (Elisevich and others 1997b). However, Cx expression does not necessarily reflect functional coupling because posttranslational modifications may alter unitary conductance, open probability, trafficking, or internalization. Hence, functional coupling analyses are indispensable. Increased astrocytic coupling has been reported by Takahashi and colleagues (2010) in a post-SE model and by Samoilova and colleagues (2003) in hippocampal slice cultures chronically exposed to bicuculline. In contrast, Xu and colleagues (2009) observed reduced coupling in the hippocampal CA1 region in a model of tuberous sclerosis complex. Human coupling studies have so far only been performed on astrocyte cultures derived from epileptic specimens (Lee and others 1995) in which enhanced coupling was found with fluorescence recovery after photobleaching.

Another approach to assess the role of GJ channels in epilepsy is the pharmacological inhibition of interastrocytic communication. Such experiments have been performed in a variety of in vivo and in vitro models of epilepsy (Bostanci and Bagirici 2006, 2007; Gajda and others 2003; Jahromi and others 2002; Kohling and others 2001; Medina-Ceja and others 2008; Perez-Velazquez and others 1994; Ross and others 2000; Samoilova and others 2003, 2008; Szente and others 2002; Voss and others 2009). Most of these studies reported aniticonvulsive effects of GJ blockade, although opposite effects were observed by Voss and colleagues (2009). In neocortical slices from patients with mTLE or focal cortical dysplasia, GJ inhibitors attenuated epileptiform activity (Gigout and others 2006). Major problems with GJ blockers are their significant side effects and poor Cx isoform specificity.

In conclusion, Cx expression studies and functional coupling analyses yield an inconsistent picture of the role of the astroglial network in the pathophysiology of epilepsy. Further work is needed to clarify this issue.

### Role of Cx Hemichannels in Epilepsy

In addition to intercellular communication, functional membrane-spanning Cx hemichannels (HCs) have been demonstrated in astrocytes (Fig. 3). These channels are nonselective and permeable for large molecules, such as ATP, glutamate, glucose, and glutathione. Under normal conditions, these channels are closed, but the open probability increases upon depolarization, altered intracellular and extracellular Ca^2+^ concentration, metabolic inhibition, or proinflammatory cytokines (Orellana and others 2013; Theis and Giaume 2012). Activated HCs might promote neuronal hyperactivity and hypersynchronization through the excessive release of ATP and glutamate, which in turn increases excitability and Ca^2+^ wave propagation (Bedner and Steinhäuser 2013). Furthermore, GJ channels and HCs are oppositely regulated by proinflammatory cytokines (Meme and others 2006; Retamal and others 2007) and may play differential roles in epilepsy. In hippocampal slice cultures exposed to bicuculline, Yoon and colleagues (2010) showed that selective inhibition of HCs by low concentrations of mimetic peptides had protective effects, while blockade of both HCs and GJs by high doses of the peptide exacerbated lesions.

### Role of Aquaporin-4 in Regulation of Excitability

Aquaporins mediate transmembrane water movements in response to osmotic gradients. Among the isoforms identified so far, aquaporin-4 (AQP4) is the predominant water channel in the brain, where it is mainly localized to astrocyte perivascular end-feet and perisynaptic processes (Papadopoulos and Verkman 2013) (Fig. 3). Moreover, AQP4 is implicated in the pathogenesis of epilepsy mainly due to its role in regulating extracellular fluid osmolarity and extracellular space (ECS) volume (Schwartzkroin and others 1998). Osmolarity-induced shrinkage of the ECS causes hyperexcitability (Chebabo and others 1995; Dudek and others 1990; Pan and Stringer 1996; Roper and others 1992), while increasing the ECS volume fraction attenuates epileptiform activity (Dudek and others 1990; Haglund and Hochman 2005; Pan and Stringer 1996; Traynelis and Dingledine 1989). In addition to ECS regulation, the spatial overlap of AQP4 with Kir4.1 in glial end-feet gave rise to the hypothesis that AQP4 may be involved in K^+^ homeostasis (Binder and others 2012).

Insight into the role of AQP4 in ECS regulation, K^+^ clearance, and excitability came from transgenic mice: AQP4-deficient (AQP4^–/–^) mice display mild ECS volume expansion as assessed by Fluorescence Recovery After Photobleaching (FRAP) and theTetraMethylAmmonium+ (TMA+) method (Binder and others 2004b; Yao and others 2008). In acute seizure models, AQP4^–/–^ mice exhibited an elevated seizure threshold but prolonged seizure duration (Binder and others 2004a; Lee and others 2012). In addition, in vivo and in situ studies using K^+^-sensitive electrodes or a fluorescent K^+^ sensor revealed impaired stimulus-induced [K^+^]_o_ clearance in AQP4-deficient mice (Binder and Steinhäuser 2006). Interestingly, K^+^ spatial buffering was enhanced in AQP4^–/–^ mice probably due to improved GJ coupling (Strohschein and others 2011). The expanded ECS volume found in AQP4-deficient mice offers an explanation for the elevated seizure threshold of these mice, while the impaired K^+^ uptake might account for the prolonged duration and increased frequency of seizures. However, the mechanistic link between AQP4 expression and K^+^ homeostasis has not been resolved yet. The view of a functional interaction between AQP4 and Kir4.1 (Nagelhus and others 1999) is not supported by studies showing AQP4-independent Kir4.1 function (Ruiz-Ederra and others 2007; Strohschein and others 2011; Zhang and Verkman 2008). Rather, astrocytic K^+^ uptake during neuronal activity might trigger AQP4-dependent water uptake and a reduction of the ECS. In turn, ECS shrinkage might increase [K^+^]_o_ and consequently further K^+^ uptake by astrocytes (Jin and others 2013; Papadopoulos and Verkman 2013).

### AQP4 Expression and Regulation in Epileptic Tissue

Ultimately, AQP4 expression was investigated in hippocampi of patients with mTLE using reverse trascrition-PCR, immunohistochemistry, and gene chip analysis (Lee and others 2004). The authors found enhanced AQP4 levels in HS but reduced expression of the dystrophin gene, which encodes the protein that is involved in anchoring AQP4 in perivascular end-feet, and speculated that polarity in astrocytic AQP4 distribution got lost. This hypothesis was subsequently confirmed with immunogold electron microscopy and Western blot analysis (Eid and others 2005). This locally restricted reduction of AQP4 was accompanied by a loss of perivascular dystrophin, indicating that AQP4 mislocalization was caused by a disrupted dystrophin complex. Similar losses of perivascular AQP4 and dystrophin were found in tissue from patients with focal cortical dysplasia (Medici and others 2011). A further indication for the involvement of AQP4 in epilepsy and for the proposed interplay between AQP4 and Kir4.1 comes from genetic studies showing that several SNPs in the KCNJ10 and AQP4 genes are associated with mTLE (Heuser and others 2010) (see also below).

In a recent study, Alvestad and colleagues (2013) explored in a kainate model whether the loss of perivascular AQP4 found in human mTLE is involved in epileptogenesis or merely represents a consequence of the condition. They demonstrated that AQP4 mislocalization precedes the chronic phase of epilepsy, suggesting that astrocytic dysfunction is of pathophysiological relevance.

## Glutamate: Transporters and Degradation

### Extracellular Glutamate Levels in TLE

The rapid clearance of excessive glutamate from the ECS and the recycling of glutamate are important processes for brain function. To prevent excitotoxic accumulation in the ECS, glutamate is taken up by astrocytes via transporters, converted to glutamine by the enzyme glutamine synthetase (GS), and shuttled back to neurons for resynthesis of glutamate (Fig. 3). Dysfunction of glutamate metabolism seems to be critically involved in the pathophysiology of epilepsy (Coulter and Eid 2012). Accordingly, glutamate and its analogs cause seizures and neuronal loss in experimental epilepsy (Ben-Ari 1985; Fremeau and others 2002; Nadler and Cuthbertson 1980; Olney and others 1972). Increased extracellular glutamate levels were found in the hippocampi of patients with mTLE (Cavus and others 2005; During and Spencer 1993). Patients with HS displayed the highest interictal glutamate levels (Petroff and others 2003). However, the source of glutamate in HS is obscure because one of the hallmarks of this pathology is the loss of glutamatergic neurons in the hippocampal CA1 region.

### Astrocytic Glutamate Uptake in Epilepsy

Uptake of glutamate from the ECS is driven by the electrochemical gradients. Five transporter isoforms have been identified, and two of them, GLAST (EAAT1) and GLT1 (EAAT2), are preferentially expressed in astrocytes (Fig. 3). The impact of astrocytic glutamate uptake became obvious from mice devoid of transporter proteins. GLT1 knockout mice displayed enhanced seizure susceptibility (Tanaka and others 1997). The pharmacological inhibition of GLT1 in the rat neocortex reduced the threshold for evoking epileptiform activity (Campbell and Hablitz 2004; Demarque and others 2004). In contrast, knockdown of GLT1 caused increased extracellular glutamate, but not seizures (Rothstein and others 1996). Mice deficient in GLAST showed no spontaneous seizures, but amygdala kindling or provoked seizures were of longer duration, were more severe, and occurred after a shorter latency (Watanabe and others 1999).

In patients with mTLE, data on the regulation of GLT1 and GLAST are inconsistent. Decreased GLT1 and GLAST levels were found by Mathern and colleagues (1999) and Proper and colleagues (2002), while Tessler and colleagues (1999) and Eid and colleagues (2004) reported no changes. In kindled rats, unchanged GLT1 and GLAST levels were described (Akbar and others 1997; Miller and others 1997; Simantov and others 1999), while decreased levels were found in pilocarpine (Lopes and others 2013) and albumin models (David and others 2009) as well as in tuberous sclerosis (Wong and others 2003). Finally, Guo and colleagues (2010) observed decreased GLAST but unaffected GLT1 expression in the hippocampus of spontaneously epileptic rats.

Together, these studies indicate that astrocytic glutamate uptake plays a crucial role in protecting neurons from hyperexcitability. However, how exactly this mechanism is disturbed in epilepsy is still under discussion.

### Regulation of GS in Epilepsy

**Figure 4.**
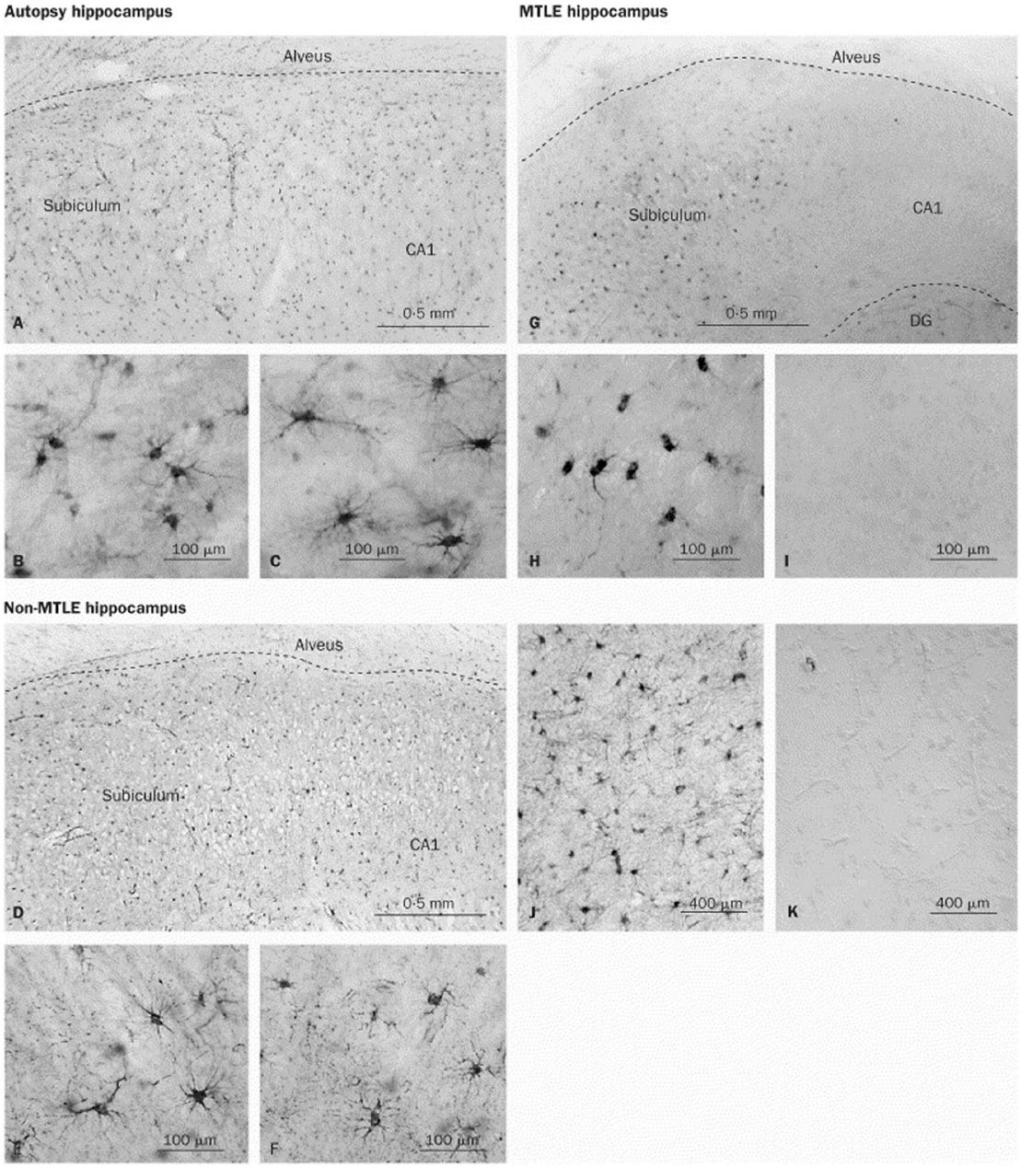
Decreased glutamine synthetase (GS) immunoreactivity in the CA1 region of patients with medial temporal lobe epilepsy (mTLE). There is a dense and even distribution of GS-positive cells in the subiculum and CA1 region of autopsy (A) and non-mTLE hippocampi (D). High-power fields of the subiculum in autopsy (B) and non-mTLE (E) hippocampi show that staining is confined to astrocytes. High-power fields of the CA1 area in autopsy (C) and non-mTLE (F) hippocampi also show many GS-positive astrocytes. In the mTLE hippocampus (G), there are many GS-positive cells in the subiculum, but the CA1 area is severely deficient in GS staining. High-power view of the subiculum in G (H) confirms the presence of staining in astrocytes, which have fewer processes than GS-positive astrocytes in the corresponding areas of autopsy (B) and non-mTLE hippocampi (E). (I) High-power view of the CA1 area in G confirms the lack of GS staining in this region. Specificity controls with GS antiserum (J) and preimmune serum (K) on adjacent sections of the non-mTLE hippocampus shown in D to F reveal no staining in K. DG = dentate gyrus. Modified and reproduced with permission from Eid and others (2004).

In the CNS, GS is predominantly expressed by astrocytes, where it converts glutamate and ammonia to glutamine (Fig. 3). Impaired GS activity plays a crucial role in the pathogenesis of mTLE (Eid and others 2013b). Thus, intrahippocampal infusion of a GS inhibitor caused recurrent seizures and neuropathological changes similar to human mTLE (Eid and others 2008; Perez and others 2012; Wang and others 2009). Moreover, decreased GS protein and enzyme activities, probably due to posttranscriptional modification (Eid and others 2013a), have been found in patients with mTLE-HS (Eid and others 2004; van der Hel and others 2005) (Fig. 4). Finally, mutations in the GS encoding gene, *GLUL*, are associated with reduced GS activity and seizures (Haberle and others 2005, 2006, 2011). One argument against a causative role for GS dysfunction in epileptogenesis, however, arises from the study by Hammer and colleagues (2008), who in a kainate model confirmed decreased GS proteins in the chronic phase but reported increased GS levels in the latent period (prior to seizure onset). The link between GS down-regulation and seizure development is based on the assumption that GS deficiency causes intracellular glutamate accumulation. This accumulation, in turn, slows down glutamate uptake, leading to increased glutamate levels in the ECS. Indeed, GS inhibition entails glutamate accumulation in hippocampal astrocytes (Perez and others 2012).

Interruption of the glutamate-glutamine cycle through GS inhibition impairs inhibitory GABAergic transmission (Liang and others 2006) but has little effect on excitatory synaptic function (Kam and Nicoll 2007). The consequences of this relationship have been studied in a model of astrocytic gliosis with GS down-regulation, which produced a deficit in inhibitory, but not excitatory, synaptic transmission. Employing voltage-sensitive dye imaging, the authors showed that these inhibitory deficits entail network hyperexcitability, which could partially be reversed by exogenously supplied glutamine. These data emphasize the importance of proper GS function for inhibitory neurotransmission and the prevention of hyperexcitability (Ortinski and others 2010).

## Astrocyte Ca^2+^ Signaling, Gliotransmitter Release, and Epileptiform Discharges in TLE

As a consequence of Ca^2+^ elevations, astrocytes can release gliotransmitters that differently modulate neuronal excitability and synaptic transmission. Some of these gliotransmitters, such as glutamate, D-serine, and ATP, have been proposed to be released through a Ca^2+^-dependent mechanism that involves vesicle exocytosis (Bezzi and others 2004; Martineau and others 2013; Parpura and Zorec 2010), lysosome exocytosis (Li and others 2008; Zhang and others 2007), or permeation through ion channel openings upon intracellular Ca^2+^ elevations (Takano and others 2005; Woo and others 2012) (Fig. 1). Gliotransmitter Ca^2+^-independent release mechanisms have been also identified and rely on the opening of large pore channels, such as anion channels and P2X7 receptors, or the reversal operation of glutamate transporters (Fellin and Carmignoto 2004; Parpura and others 2004). While we cannot exclude that Ca^2+^-dependent and Ca^2+^-independent gliotransmitter release mechanisms coexist and are operative under different conditions, from the presence of astrocyte Ca^2+^ elevations, we can infer that gliotransmitter release is operative in these cells. Below, we discuss recent studies that described an enhancement of Ca^2+^ signals in astrocytes and a synchronization of neuronal activities by gliotransmitter release during epileptiform activity.

### Astrocyte Ca^2+^ Signals

If astrocytes contribute to the generation of seizures, it is expected that not only neurons but also astrocytes are hyperactive in epileptic brain circuits. Recent studies provided evidence that during epileptiform activities, Ca^2+^ signaling in astrocytes is indeed enhanced. For example, in rat hippocampal slices in which network excitability was enhanced by reducing external Mg^2+^ and by blocking inhibitory synapses with the GABA_A_ receptor antagonist picrotoxin, both the number of astrocytes displaying Ca^2+^ oscillations and the frequency of Ca^2+^ peaks were significantly increased (Fellin and others 2006). The increased Ca^2+^ oscillation frequency correlated with an increased frequency of the slow inward currents (SICs) observed under these conditions in pyramidal neurons. In vivo studies also showed that epileptiform activities induced by various pharmacological treatments were associated with an increased frequency of Ca^2+^ oscillations in cortical astrocytes (Ding and others 2007; Tian and others 2005). Interestingly, in the same in vivo models of epilepsy, the increase in Ca^2+^ oscillation frequency was reduced by the systemic administration of anticonvulsant drugs, such as valproate, gabapentin, and phenytoin (Tian and others 2005). Consistent with an increased activity of astrocytes in the epileptic brain, the astrocytic expression of the metabotropic glutamate receptors (mGluRs), which mediates the Ca^2+^ response of astrocytes to synaptically released glutamate, is increased in animal models of TLE (Aronica and others 2000; Ulas and others 2000).

Altogether, these data indicate that a significant correlation exists between an increase in astrocyte Ca^2+^ oscillations and the emergence of epileptiform activities and suggest that astrocytes may contribute to the generation of epileptic discharges.

### Gliotransmitter Release

An experimental observation that provided further support to the potential role of astrocytes and Ca^2+^-dependent gliotransmission in the generation of epileptiform activities was obtained in young rat hippocampal slices (Fellin and Carmignoto 2004). In these experiments, Ca^2+^ elevations evoked in astrocytes by various stimuli that activated G protein–linked receptors, including the synaptic release of glutamate induced by Schaffer collateral stimulation, were followed by the generation in pyramidal CA1 neurons by SICs (Fellin and others 2004). These events appear to originate from a Ca^2+^-dependent release of glutamate from astrocytes as they were insensitive to the block of action potential–dependent glutamate release from neurons by tetrodotoxin (TTX); could be induced by photolysis of a Ca^2+^-caged compound loaded into individual astrocytes; and were mediated by extrasynaptic, high-affinity N-methyl D-aspartate receptor 2B (NR2B)–containing N-methyl-D-aspartate receptors (NMDARs) (Fellin and Carmignoto 2004). Similar events have been subsequently observed in different brain regions, including the thalamus (Parri and others 2001), nucleus accumbens (D’Ascenzo and others 2007; Fellin and others 2007), olfactory bulb (Kozlov and others 2006), brain stem (Reyes-Haro and others 2010), spinal cord (Bardoni and others 2010), and neocortex (Ding and others 2007). In relation to a potential role of astrocytic glutamate in epileptiform activity, it is worth stressing here that SICs can occur with a high level of synchrony among two nearby pyramidal neurons (Angulo and others 2004; Fellin and Carmignoto 2004). Given that excessive neuronal synchronization in local networks is one of the hallmarks of epileptic disorders, SICs may be involved in the generation of epileptiform activity. In addition, the observation that SICs can depolarize the neuronal membrane to the action potential discharge threshold (Fellin and others 2006; Pirttimaki and others 2011) suggests that astrocytic glutamate, initially acting on one or two neurons, may enlarge its action to synaptically connected neurons and promote focal epileptiform discharges by recruiting large neuronal populations into synchronous bursts. Consistent with this hypothesis, confocal microscope Ca^2+^ imaging experiments, which allow us to evaluate neuronal network activities by monitoring simultaneously the Ca^2+^ signal dynamics from tens of neurons, revealed that astrocytic glutamate triggers simultaneous NMDA receptor–mediated Ca^2+^ increases in groups of CA1 neurons, an event that was termed a “domain response” (Fellin and others 2004). It is important to underline that the proconvulsant action of astrocytic glutamate may have more impact in regions that are rich with recurrent axon collaterals, such as the hippocampus and other limbic structures, where it may represent a nonneuronal mechanism that favors neuronal synchrony (Carmignoto and Fellin 2006).

The hypothesis that the activation-induced glutamate release from astrocytes is directly involved in the generation of epileptic events was specifically investigated in a number of in vitro and in vivo studies. A study by Tian and colleagues (2005), using different slice models of chemically induced epilepsy, proposed the paroxysmal depolarizing shifts (PDSs) that characterize interictal epileptiform events to correspond in large part to the depolarizing events generated by SICs and thus result from the release of glutamate from astrocytes, which were strongly activated under these experimental conditions. In favor of an astrocytic origin of PDSs, the authors showed that a large majority of these events were insensitive to action potential inhibition by the Na^+^ channel blocker TTX. These observations were, however, at variance with results obtained in a number of studies reporting a sensitivity to TTX of interictal discharges (Fellin and others 2006; Gomez-Gonzalo and others 2010; Perreault and Avoli 1991; Stasheff and others 1993). Also, differences in the sensitivity of PDSs and SICs to NMDA receptor blockers—that is, D-AP5 abolished SICs, but not PDSs—render unlikely that PDSs coincide with astrocyte-mediated SICs. It thus appears that PDSs and SICs share similar kinetics but are distinct events generated by two different cellular sources (D’Ambrosio 2006; Wetherington and others 2008). Subsequent studies clarified that astrocyte Ca^2+^ signals leading to glutamate release cooperate with neurons in the generation of an epileptogenic focus rather than represent a direct cause of the epileptic discharge. In a young rat cortical slice model of TLE, Gomez-Gonzalo and colleagues (2010) showed that local NMDA applications to entorhinal cortex layer V to VI neurons, in the presence of the proconvulsant 4-aminopyridine and low external Mg^2+^, can trigger focal seizure–like discharges. The authors observed that two subsequent, but not single, NMDA pulse stimulations evoked both a synchronous and intense firing discharge in a small group of neurons and early Ca^2+^ elevations in astrocytes. This local response was regularly followed by a seizure-like event that emerged at the focus with a few seconds’ delay and later propagated to distant brain regions. The early Ca^2+^ elevation in astrocytes represented a central event in NMDA-induced seizure generation. Indeed, after a drastic reduction of the Ca^2+^ increase by the Ca^2+^ chelator 1,2-bis(o-amino-phenoxy)ethane-N,N,N’,N’-tetraacetic acid (BAPTA), by patching individual astrocytes with a BAPTA-containing pipette that allows the spreading of BAPTA throughout the astrocyte syncytium, double NMDA stimulation, which normally triggered a seizure discharge, became unsuccessful. Importantly, weak NMDA stimulation, composed of a single NMDA pulse that failed to trigger both Ca^2+^ signals in astrocytes and seizures, became successful when it was coupled with a stimulus that triggered Ca^2+^ elevations in astrocytes, such as L- Threonyl- L- phenylalanyl- L- leucyl- L- leucyl- L-argininamide (TFLLR), that is, a peptide that activates thrombin protease-activated receptor 1. Of note, this receptor is highly, if not selectively, expressed in astrocytes of the entorhinal cortex, and it is known to mediate glutamate release in these cells (Fellin and others 2004). As a whole, these data suggest that astrocytic Ca^2+^ signals induced in astrocytes by neurons through NMDA applications evoke the release of gliotransmitters, such as glutamate and possibly D-serine, which enhanced NMDA receptor activation. This, in turn, leads to the recruitment of a critical mass of neurons that, in turn, generates a focal seizure discharge (Gomez-Gonzalo and others 2010).

The initiation of a seizure discharge at the epileptogenic focus may thus be due to the intense firing of a critical mass of neurons in a recruitment process that involves an excitatory loop between neurons and astrocytes. When astrocytes are consistently engaged by an episode of hyperactivity in a group of neurons, they signal back by a Ca^2+^-dependent gliotransmitter activity that increases the basic excitability of brain microcircuits. If this feedback signal operates on a brain network that is abnormally hyperactive as a consequence of trauma, high temperature, or genetic defects, it will contribute to drive neurons towards the seizure discharge threshold.

A recent study in *Drosophila* provides further support to this conclusion. The study by Melom and Littleton (2013) reported that in the *Drosophila* brain, a mutation of the glial-specific K^+^-dependent Na^+^/Ca^2+^ exchanger in brain glia leads to a series of defective features in these cells, including an increase in Ca^2+^ basal levels and a suppression of microdomain Ca^2+^ oscillations that are normally observed in controls. Most importantly, these *zyd* mutants exhibit an enhancement of seizure susceptibility to increased temperature. Given that these glial cells form in the *Drosophila* brain a honeycomb network, in a fashion similar to the mosaic distribution of astrocytes in the mammalian brain, and also that they encapsulate individual neuronal somata similarly to the typical anatomic features of astrocytic processes, these glial cells are in a privileged position to exchange with neurons’ functional signals. The authors indeed suggested that in *zyd* mutants, glia exhibiting high Ca^2+^ basal levels respond to a stressful environmental stimulus with an additional Ca^2+^ increase that, through glia-to-neuron signaling, initiates a seizure-inducing process. Consistent with the role of glia Ca^2+^ elevations in seizure generation, they induced a selective expression of the heat-activated TRPA1 channels in glia and observed a strong, immobilizing seizure within seconds of TRPA1 stimulation by a temperature shift to 30°C. Although it remains to be fully investigated how glia signal to neurons in this model, this study provides further support for a direct role of glia in seizure generation.

In addition to enhancing the excitability of single neurons and favoring neuronal synchronies, the release of glutamate from astrocytes contributes to the delayed neuronal death that characterizes chronic epileptic conditions (Ding and others 2007). During SE, astrocytes are highly activated, and the consequent glutamate release can trigger neuronal damage through an excessive activation of NR2B-containing NMDA receptors. The inhibition of astrocyte Ca^2+^ signals by the mGluR antagonist 2-Methyl-6-(phenylethynyl)pyridine (MPEP) as well as the block by ifenprodil of extrasynaptic 2B subunit–containing NMDA receptors, that is, the neuronal targets of astrocytic glutamate, reduce the delayed neuronal death that characterizes this pathological condition (Ding and others 2007). Consistent with the role of extrasynaptic NR2B-containing NMDA receptors in cell death during SE is the finding that activation of this receptor subtype results in mitochondrial dysfunction and stimulates both dephosphorylation of the cyclic adenosine monophosphate response element binding protein and neuronal death following hypoxic ischemic insults or head injuries (Hardingham and others 2002).

## Role of Astrocytes in Absence Seizures

### Astrocytic GABA Transporters

Classically, epilepsy is viewed as originating either from enhanced glutamatergic transmission, decreased GABAergic transmission, or both. Thus, the mutations in GABA_A_ receptor genes of patients with absence seizures (Kananura and others 2002; Lachance-Touchette and others 2011; Maljevic and others 2006; Macdonald and others 2010; Wallace and others 2001) have always been interpreted as leading to a widespread loss of function in GABA_A_R-mediated synaptic transmission. However, in transgenic mice carrying one of this human GABA_A_R point mutations (i.e., R43Q in the γ subunit) (Wallace and others 2001), abnormalities in GABAergic transmission (i.e., decreased inhibitory postsynaptic current [IPSC] frequency) are present in cortical but not in thalamic reticular or thalamocortical neurons (Tan and others 2007). Indeed, many pieces of independent evidence indicate that in thalamocortical neurons of sensory thalamic nuclei of many absence epilepsy models, the GABAergic function is not decreased but is either increased or unchanged. In particular, 1) in Genetic Absence Epilepsy Rats from Strasbourg (GAERS), a well-established genetic absence model (Danober and others 1998), thalamocortical neurons show rhythmic bursts of GABA_A_ inhibitory postsynaptic potentials (IPSPs) during absence seizures (Pinault and others 1998); 2) the thalamic injection of penicillin (Kostopoulos 2000) or bicuculline (Steriade and Contreras 1995) fails to elicit absence seizures; 3) in many mouse models, there is no change or an increased phasic GABA_A_R-mediated inhibition (i.e., IPSPs or IPSCs) in thalamocortical neurons compared to their respective nonepileptic control strains (Bessaih and others 2006; Caddick and others 1999; Cope and others 2009; Tan and others 2008); 4) GABA levels in the thalamus of GAERS are higher than in the nonepileptic control strain (Richards and others 1995); and 5) GABA_B_R agonists induce absence seizures in naïve animals and aggravate them in different models of this nonconvulsive epilepsy (Aizawa and others 1997; Danober and others 1998; Snead 1992). Importantly, drugs that increase GABA levels, that is, vigabatrin and tiagabine, induce absence seizures in animals and humans as well as aggravate them in animal models of, and in patients suffering from, absence epilepsy (Danober and others 1998; Ettinger and others 1999; Hosford and Wang 1997; Panayiotopoulos 2001; Perucca and others 1998).

In line with these findings, tonic GABA_A_ inhibition in thalamocortical neurons is increased in mouse and rat genetic and pharmacological models of absence epilepsy, including GAERS, stargazer, and lethargic and succinic semialdehyde dehydrogenase knockout animals as well as in the γ-hydroxybutyric acid (GHB) and 4,5,6,7-tetrahydroisoxazolo-[5,4-C]pyridine-3-ol (THIP) models (Cope and others 2009; Errington and others 2011b). Moreover, absence seizures in GAERS are blocked by the thalamic injection of a GABA_A_R δ subunit–specific antisense oligodeoxynucleotide and cannot be induced in GABA_A_R δ-subunit knockout mice (Cope and others 2009). Together, these data suggest a potential therapeutic role for inverse agonists at perisynaptic/extrasynaptic δ-GABA_A_Rs in absence epilepsy (Errington and others 2011a).

**Figure 5.**
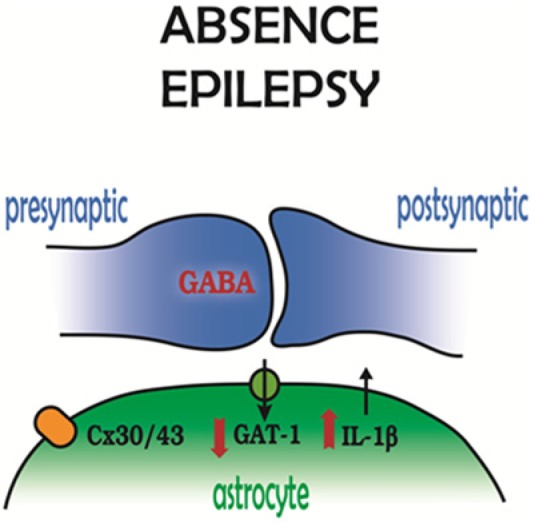
Astrocytic molecular players in absence epilepsy. GABAergic synapses in the sensory thalamus show a decreased function of the astrocytic GABA transporter GAT-1, while in the somatosensory cortex, interleukin-1β levels are increased. Astrocytic thalamic gap junctions, that is, connexin 30 and connexin 43, also contribute to absence seizure generation. Modified and reproduced with permission from Crunelli and Carmignoto (2013).

The increased tonic GABA-A current in thalamocortical neurons of genetic mouse and rat models does not result from an enhanced activity of perisynaptic/extrasynaptic δ subunit–containing GABA_A_Rs, but it is due to a loss of function of one of the GABA transporters, GAT-1 (Cope and others 2009) (Fig. 5), which in the thalamus of both humans and rodents is exclusively located in astrocytes (Borden 1996; De Biasi and others 1998; Pow and others 2005). This conclusion is based both on indirect evidence (i.e., measurements of the tonic GABA_A_ current in GAERS and stargazer mice) (Cope and others 2009) and direct evidence showing that the GABA transporter current measured from patch-clamped astrocytes in GAERS thalamic slices is not affected by NO-711 (a selective blocker of GAT-1) but is abolished by SNAP5114, a selective blocker of GAT-3 (Pirttimaki and others 2012).

Interestingly, the thalamic expression of GAT-1 in GAERS and stargazer mice is similar to that of their respective nonepileptic control strains, and only a silent mutation is present in the GAT-1 gene of these two genetic models (Cope and others 2009). So, it may be possible that the inability of GAT-1 to regulate GABA levels is because this transporter remains as an immature intracellular protein or, alternatively, that the phosphorylation of GAT-1, and thus its function, is compromised. Moreover, one might expect GAT-3, the only other GABA transporter in the thalamus (Borden 1996; De Biasi and others 1998; Pow and others 2005), to compensate for the malfunctioning GAT-1, but this is not the case. It might also be that GAT-3 is located far away from the perisynaptic/extrasynaptic GABA_A_Rs that are responsible for the tonic GABA_A_ current, although a recent investigation concluded that GAT-1 is “primarily localized near GABAergic synapses whereas GAT-3 is localized both near and far away from synapses” (Beenhakker and Huguenard 2010). Clearly, more direct studies investigating the relative position of these transporters with respect to synaptic and perisynaptic/extrasynaptic GABA_A_Rs are necessary to resolve this issue.

### Astrocyte-Neuron Signaling in Absence Seizures

Slow outward currents (SOCs) are the characteristic signatures of GABAergic astrocyte-to-neuron signaling (Kozlov and others 2006). Possibly as a consequence of reduced GAT-1 activity in the thalamus, some properties (i.e., amplitude, rise time, and decay time) of SOCs are altered in GAERS thalamocortical neurons (Pirttimaki and others 2011, 2012). At present, one cannot ascribe a precise mechanistic significance for these changes in thalamic SOCs to the pathophysiological processes underlying absence seizures. Nevertheless, it is interesting that vigabatrin, which elicits and/or exacerbates absence seizures in animals and humans (see above), can increase the frequency of SOCs in thalamic neurons (Jimenez-Gonzalez and others 2011).

Importantly, no differences are observed in the properties of SICs, the characteristic signature of glutamatergic astrocyte-to-neuron signaling (Angulo and others 2004; Fellin and others 2004), between GAERS prior to seizure onset and age-matched nonepileptic rats (Pirttimaki and others 2012). Moreover, no change in astrocytic glutamate transporters has been reported at this age in the thalamus of this genetic model (Dutuit and others 2002).

It is now well established that, contrary to the classic view, absence seizures are not generalized from their very start, but there is an “initiation site” in a localized cortical region both in experimental models (Meeren and others 2002; Polack and others 2007) and in humans (Bai and others 2010; Holmes and others 2004; Moeller and others 2010; Westmijse and others 2009). Thus, it is important that future studies investigate whether similar abnormalities to those present in the activity of GAT-1 and SOCs are also present in the cortical “initiation site” of rat genetic models and humans suffering from this form of epilepsy. Indeed, in cortical neurons, one might expect to see alterations in SICs because glutamate uptake is reduced in this brain region of preseizure GAERS (Touret and others 2007) as a result of a decreased expression of the astrocytic glutamate transporters GLT1 and GLAST (Dutuit and others 2002).

### Other Astrocytic Abnormalities in Absence Seizures

Another recent finding that stresses the potential role of astrocytic abnormalities in the expression of absence seizures is the selective induction of IL-1β in activated astrocytes within the cortical “initiation site” of absence seizures (i.e., the peri-oral region of the somatosensory cortex) (Fig. 5), but not in other cortical regions or in the thalamus of GAERS prior to seizure onset (Akin and others 2011). This enhanced cortical expression of IL-1β is not simply an epiphenomenon because the systemic injection of a specific blocker of IL-1β synthesis drastically decreases absence seizures in this model (Akin and others 2011) and the injection of an IL-1β inducer increases the number of absence seizures in WAG/Rij rats (Kovács and others 2006), another well-established model of absence epilepsy (Coenen and Van Luijtelaar 2003). Unfortunately, no data are available on the levels of IL-1β in humans before or after the onset of absence seizures.

Finally, it is important to note that the ability of the GJ blocker carbenoxolone to drastically reduce absence seizures in both rat (WAG/Rij) and mouse (lethargic) models when it is injected intrathalamically or systemically (Gareri and others 2005) (Fig. 5), respectively, has been mainly explained as resulting from a block of neuronal GJs. While strong evidence exists for neuronal GJs in the thalamus (Hughes and others 2004, 2011; Landisman and others 2002; Nagy and Rash 2000; Söhl and others 2005), both the reticular thalamic nucleus and the sensory thalamic nuclei also exhibit strong immunoreactivity for the astroglia-specific Cx30 and Cx43 (Nagy and Rash 2000) (Fig. 3). Thus, astrocytic GJs are also likely to play a role in the antiabsence effect of carbenoxolone and in the mechanisms underlying the expression of absence seizures (Fig. 5).

## Conclusions

The findings reviewed here highlight novel key elements of astrocytic involvement in epilepsy, in particular, abnormalities in the astrocyte control of extracellular K^+^, Ca^2+^ signaling, and gliotransmitter release in mTLE and abnormalities in GAT-1, Cx30- and Cx43-based GJs, and the IL-1β pathway in absence seizures. This shift in research from neurons to astrocyte-neuron interactions (Crunelli and Carmignoto 2013; Steinhäuser and others 2012) has allowed us to unravel novel mechanisms underlying the generation of seizures (Baulac and Pitkanen 2009). These novel astrocyte-based targets should pave the way to a rational discovery program for the development of fourth-generation antiepileptogenic drugs with increased efficacy that many expert groups have identified as a key priority for the improved clinical management of convulsive and nonconvulsive epilepsy (Baulac and Pitkanen 2009; Galanopoulou and others 2012; Löscher and Schmidt 2011; Simonato and others 2012).
